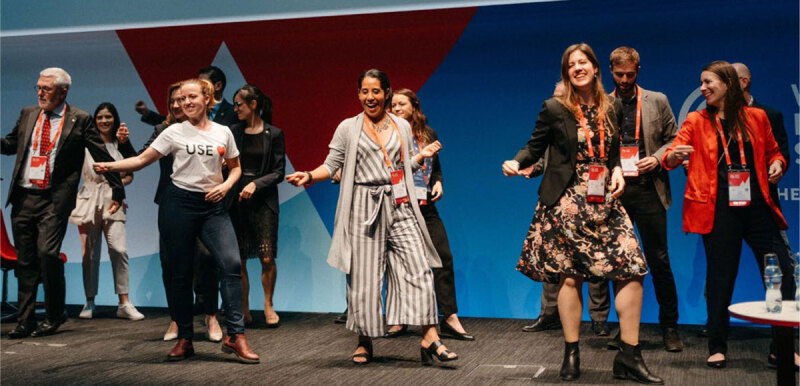# More People, More Active, More Often for Heart Health – Taking Action on Physical Activity

**DOI:** 10.5334/gh.1308

**Published:** 2024-05-03

**Authors:** Trevor Shilton, Adrian Bauman, Birgit Beger, Anna Chalkley, Beatriz Champagne, Martina Elings-Pers, Billie Giles-Corti, Shifalika Goenka, Mark Miller, Karen Milton, Adewale Oyeyemi, Robert Ross, James F. Sallis, Kelcey Armstrong-Walenczak, Jo Salmon, Laurie P. Whitsel

**Affiliations:** 1 World Heart Federation, AU; 2 Sydney University School of Public Health, AU; 3 World Heart Federation, BE; 4 European Heart Network, BE; 5 Faculty of Life Sciences and Health Studies, University of Bradford, UK; 6 Centre for Applied Education Research, Wolfson Centre for Applied Health Research, Bradford Royal Infirmary, UK; 7 Coalition for Americas’Health, US; 8 World Heart Federation, SE; 9 Swedish Heart Lung Foundation, SE; 10 Centre for Urban Research, RMIT University, AU; 11 Telethon Kids Institute, AU; 12 Public Health Foundation of India, IN; 13 Centre for Chronic Disease Control, IN; 14 World Heart Federation, UK; 15 Centre for Cardiovascular Science, University of Edinburgh, UK; 16 Norwich Medical School, University of East Anglia, UK; 17 College of Health Solutions, Arizona State University, US; 18 Queen’s University School of Kinesiology and Health Studies, CA; 19 Herbert Wertheim School of Public Health, University of California, US; 20 Mary MacKillop Institute for Health Research, Australian Catholic University, AU; 21 World Heart Federation, CH; 22 Institute for Physical Activity and Nutrition, Deakin University, AU; 23 American Heart Association, US; 24 Physical Activity Alliance, US

**Keywords:** physical activity, world heart federation, global health policy

## Abstract

Physical inactivity is a leading contributor to increased cardiovascular morbidity and mortality. Almost 500 million new cases of preventable noncommunicable diseases (NCDs) will occur globally between 2020 and 2030 due to physical inactivity, costing just over US$300 billion, or around US$ 27 billion annually (WHO 2022). Active adults can achieve a reduction of up to 35% in risk of death from cardiovascular disease. Physical activity also helps in moderating cardiovascular disease risk factors such as high blood pressure, unhealthy weight and type 2 diabetes. For people with cardiovascular disease, hypertension, type 2 diabetes and many cancers, physical activity is an established and evidence-based part of treatment and management. For children and young people, physical activity affords important health benefits. Physical activity can also achieve important cross-sector goals. Increased walking and cycling can reduce journeys by vehicles, air pollution, and traffic congestion and contribute to increased safety and liveability in cities.

## Executive Summary

As human beings, historically we were required to be physically active to survive and to go about our daily lives. However, through recent generations trends such as growth in motorised transport, sedentary work, labour-saving devices, urbanization, and screen-based recreation have contributed to more sedentary lifestyles. As a result, globally, 28 per cent of adults and more than three in four adolescents (81%) do not meet physical activity guidelines.

Physical inactivity is a leading contributor to increased cardiovascular morbidity and mortality. **Almost 500 million new cases of preventable noncommunicable diseases (NCDs) will occur globally between 2020 and 2030 due to physical inactivity, costing just over US$300 billion, or around US$ 27 billion annually (WHO 2022)**.

Active adults can achieve a reduction of up to 35% in risk of death from cardiovascular disease. Physical activity also helps in moderating cardiovascular disease risk factors such as high blood pressure, unhealthy weight and type 2 diabetes. Further, active adults can experience reduced risk of some cancers and of type 2 diabetes, and better mental health, sleep, and cognitive function. For people with cardiovascular disease, hypertension, type 2 diabetes and many cancers, physical activity is an established and evidence-based part of treatment and management. For children and young people, physical activity affords important health benefits. Risk factors for chronic health conditions, such as atherosclerosis, have been found in children as young as 10 years of age.

Physical activity can also achieve important cross-sector goals. Increased walking and cycling can reduce journeys by vehicles, and thereby can reduce air pollution, reduce traffic congestion, and contribute to increased safety and liveability in cities and communities.

This policy brief includes WHF physical activity recommendations on effective interventions for increasing population levels of physical activity summarised in Tables 7, 10. These can be adapted, at country and jurisdictional level, with consideration of community needs, culture, geography and the social and economic determinants of physical inactivity. The overarching recommendation is that “*All nations develop and implement a comprehensive National Physical Activity Policy, with implementation supported by a funded action plan”*. Supporting recommendations illustrate a comprehensive range of policy and practice recommendations and actions for physical activity, based on evidence of effectiveness, and organised around the WHO Global Action Plan on Physical Activity, 2018–2030 (WHO GAPPA) four strategic policy areas – Active Societies, Active Environments, Active People and Active Societies (WHO, 2018).

Physical activity strategies are relevant in all countries, but particularly in low-resource circumstances in low- and middle-income countries, and in environments where poverty, health literacy, remoteness and other factors may otherwise impact effectiveness or reduce access.

**Figure:**
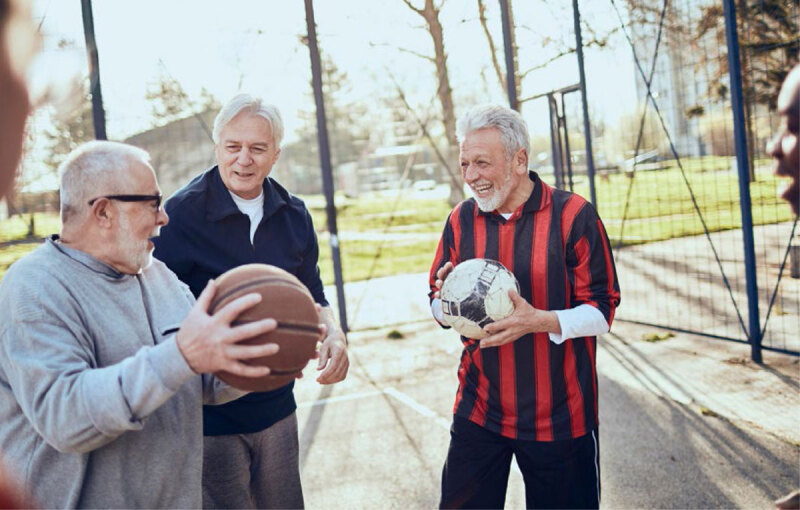


## What is physical activity?

**Throughout, we refer to physical activity, moderate physical activity and other terms to describe physical activity. The following definitions provide context for the types of physical activity referred to in our recommended actions**.

**Physical activity** is any bodily movement produced by skeletal muscles that results in energy expenditure (Caspersen et al., 1985). Physical activity can be accumulated across work, transportation, occupational, domestic and leisure time, which are referred to as ‘domains’ of physical activity.

**Light physical activity** includes activities ranging between 1.5 and <3.0 METs (metabolic equivalents), which represents activities with energy cost less than 3 times an individual’s energy expenditure at rest. Light physical activities include walking at a slow or leisurely pace, bathing, or other incidental activities that do not result in a substantial increase in heart rate or breathing rate (WHO 2020(1)).

**Moderate physical activity** includes activities ranging between 3 and <6 METS (between 3 and less than 6 times the energy expenditure at rest). On a scale relative to an individual’s personal capacity, moderate-intensity physical activity is usually a 5 or 6 on a scale of 0–10 (WHO 2020(1)). Moderate intensity activities require more oxygen consumption than light activities. People participating in moderate physical activity should be able to conduct a conversation without getting short of breath. Moderate physical activities include walking briskly, dancing, raking leaves and vacuuming.

**Vigorous intensity activities** are defined as activities ≥ 6 METS. Vigorous activities require the highest amount of oxygen consumption to participate in the activity. They include activities such as running (5 mph/8 km/h or more), swimming, heavy gardening such as digging, and jumping rope.

**Muscle strengthening activity** includes physical activity and exercise that increases skeletal muscle strength, power, endurance, and mass. This includes lifting weights, strength training, resistance training, or muscular strength and endurance exercises (WHO 2020(1)).

**Exercise** is a subcategory of physical activity. Exercise includes light, moderate and vigorous physical activity undertaken during leisure time and through sport and recreation, usually in a formal setting such as sport or a gymnasium. Exercise is planned, structured and repetitive and undertaken for the pursuit of improving fitness, excellence in performance, or health.

**Sedentary behaviour** is defined as engaging in low energy expenditure (≤ 1.5 METs) while sitting, reclining or lying during waking hours (Tremblay et al., 2017).

**Physical inactivity** is doing insufficient physical activity to meet current physical activity guidelines.

## Physical activity and cardiovascular health

### Physical activity and primary prevention of cardiovascular disease

Physical inactivity is an important risk factor for cardiovascular disease. Evidence indicates there is an inverse dose-response relationship between physical activity and cardiovascular disease and mortality risk (Lee et al., 2012).

In other words, the less physical activity, the greater risk of death from cardiovascular disease. The World Health Organization reports that about 7–8% of all cases of cardiovascular disease could be prevented if more people were physically active (WHO 2022). Indeed, by 2030, WHO estimates around 500 million new cases of preventable noncommunicable diseases (NCDs) will occur globally between 2020 and 2030 due to physical inactivity, costing US$ 27 billion annually (WHO 2022).

Evidence of the association between physical activity and cardiovascular disease was reported through epidemiological (‘population’) studies as early as the 1950s. Jerry Morris and colleagues reported that active bus conductors were protected against heart disease in comparison with inactive bus drivers (Morris et al. 1953, Morris & Crawford 1958).

Systematic reviews and meta-analyses have confirmed that moderate-to-vigorous physical activity (MVPA) decreases the likelihood of someone developing and suffering from coronary artery disease. Engaging in physical activity at a level that achieves or exceeds physical activity guidelines is associated with around a 35% reduction in risk of death from cardiovascular disease (Nocon et al., 2008). This is also reflected in recent systematic reviews conducted as part of global and national physical activity guideline processes. For example, the United Kingdom Chief Medical Officers’ Physical Activity Guidelines conclude that physical activity reduces the risk of cardiovascular disease by 35% in adults and older adults (Department of Health and Social Care, 2019). The WHO has estimated physical inactivity to be the cause of approximately 30% of ischaemic heart disease (WHO, 2010).

Light physical activity (LPA) is also associated with health benefits. Ekelund and colleagues (2019) examined the dose–response associations between device-measured LPA (accelerometry) and all-cause mortality in 36,383 adults with a mean age of 63 years with a mean follow-up of 5.8 years. The novel finding was that device-measured LPA was associated with a substantially reduced risk of death in a dose–dependent manner (i.e. the more LPA undertaken, the lower the risk of dying). This finding is consistent with the meta-analysis of Chastin and colleagues (Chastin et al., 2019) who reported that a doubling of the time spent in LPA was associated with a 29% reduction in mortality.

### Sedentary behaviour and ill-health

Sedentary behaviour is detrimental to health when undertaken for long periods and when it is undertaken as a replacement for physical activity.

Accumulated as well as prolonged sedentary behaviour has been shown to be associated with higher risk of obesity and poor metabolic health (Chastin et al., 2015; Brocklebank et al., 2015), type 2 diabetes, cardiovascular disease, some cancers, and premature all-cause mortality (WHO 2020, Lynch et al., 2010). The poor-health effects of sedentary behaviour is highest in those with low levels of moderate to vigorous physical activity (WHO 2020).

**Figure:**
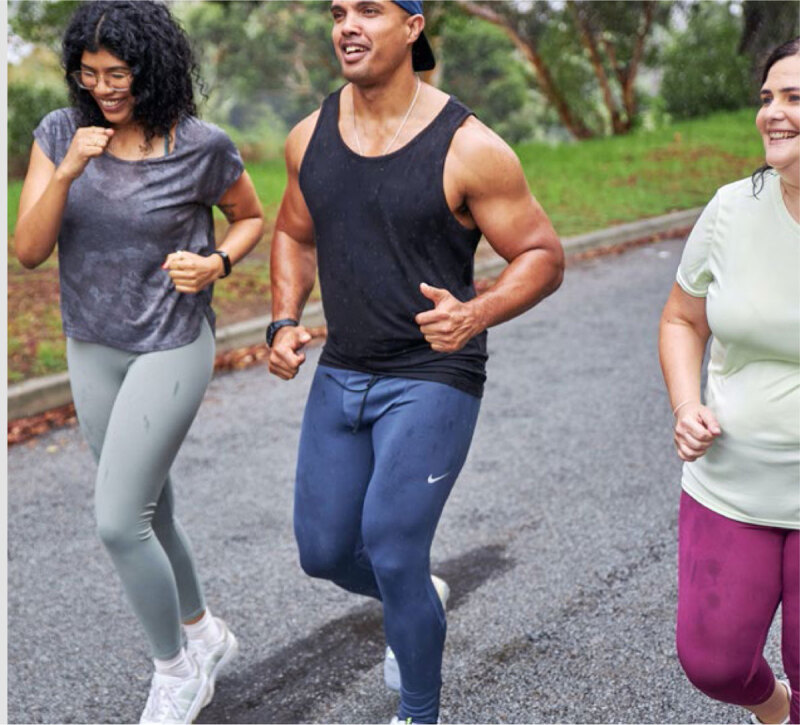


### Physical activity and impact on other CVD risk factors

In addition to the independent impact of physical activity on cardiovascular disease itself, physical activity favourably impacts multiple modifiable risk factors. Most important among these are reductions in high blood pressure, and helping to maintain a healthy weight, and primary prevention of type 2 diabetes (Al Mallah et al., 2018). Regular physical activity also contributes to reductions in blood levels of triglycerides (a type of fat), lower insulin levels (a hormone that relates to blood sugars) and increased high density lipoprotein (HDL; a blood factor that helps prevent fatty plaque build-up in arteries) (USDHHS 2018). These are significant risk factors for many cardiovascular conditions.

For people living with hypertension (clinically high blood pressure) physical activity lowers blood pressure, it decreases mortality from cardiovascular disease, reduces disease progression, and improves physical function and health-related quality of life (WHO 2020).

**Figure:**
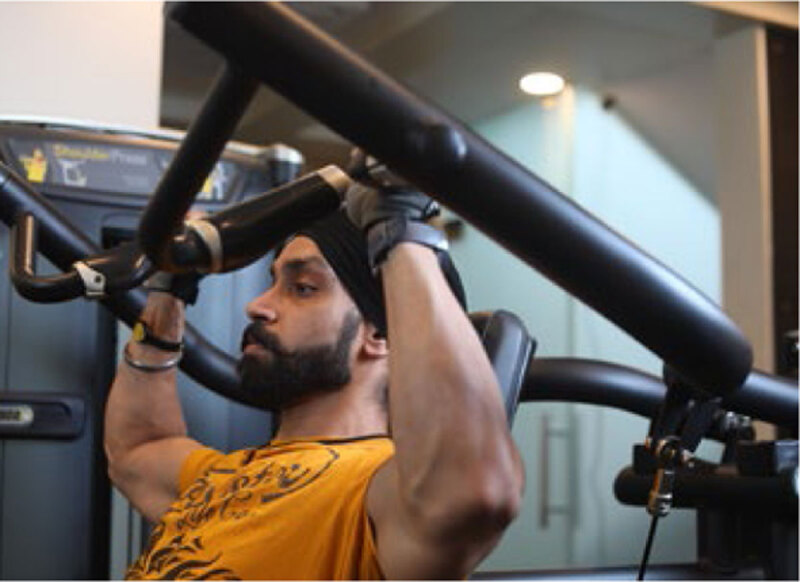


### Physical activity for people living with heart disease

For people living with heart disease participation in physical activity at recommended levels and reducing sedentary behaviours such as sitting and watching television, are helpful in maintaining good health and in reducing the risk of further cardiac events (for example a heart attack).Exercise training as part of secondary prevention and cardiac rehabilitation can reduce progression of heart disease, assist in management of cardiovascular risk factors and reduce likelihood of further cardiac events. Physical activity during recovery can help support mental health, an early return to work, domestic and other duties, and can foster development of self-management skills.

People living with heart disease should consult with a physical activity specialist or health-care professional for advice on the types and amounts of activity appropriate for their individual needs, abilities, functional limitations/complications, medications, and overall treatment plan.

**Figure:**
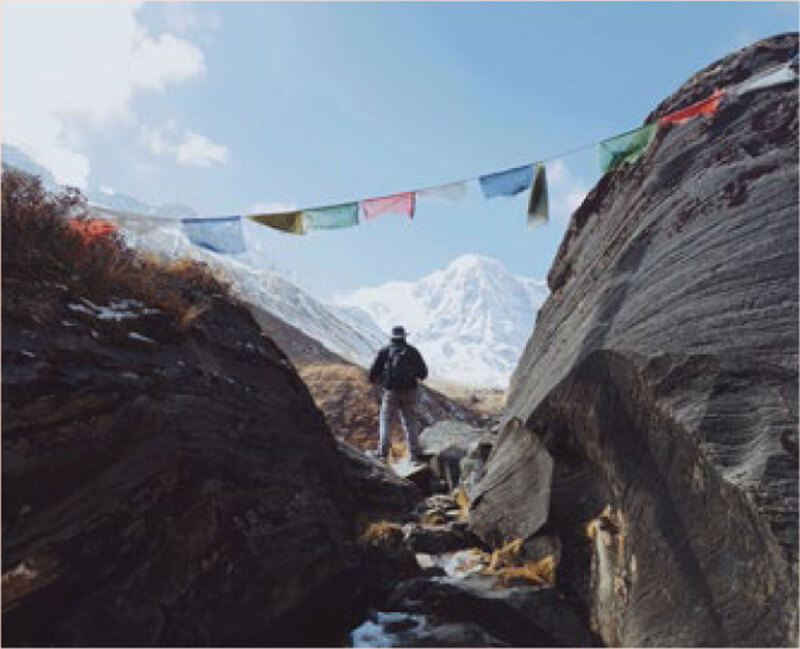


### Physical activity and cardiac rehabilitation

Cardiac rehabilitation is a category of secondary prevention. It can be defined as a multi-factorial and comprehensive intervention designed for people who have had a cardiac event and is designed to limit the physiological, psychological and social effects of cardiovascular disease, manage symptoms, restore functions, and reduce the risk of future cardiovascular events (Piepoli et al., 2017).

There is strong evidence that exercise training, as part of cardiac rehabilitation, following myocardial infarction (damage and death of heart tissue) and/or coronary revascularisation (surgery of the blood vessels of the heart) decreases mortality and morbidity, reduces hospital admissions and increases health-related quality of life (Anderson et al., 2016; Dalal et al., 2015). Increasing physical activity and reducing sedentary behaviour is helpful in reducing the risk of further cardiac events (BACPR 2017). Finally, cardiac rehabilitation can help support an early return to work, domestic and other duties and can foster development of self-management skills (Yohannes et al., 2010).

Cardiac rehabilitation guidelines globally, all recommend exercise training to aid recovery (Thomas et al., 2018; BACPR 2017; National Heart Foundation of Australia, 2019; Woodruffe et al., 2014). Patients recovering from myocardial infarction should receive an individualised exercise assessment. This combined with consideration of diagnosis, risk factors, functional capability and participant preferences, better enables provision of a tailored exercise programme of appropriate and increasing intensity, frequency and duration.

Exercise should occur as part of a comprehensive cardiac rehabilitation programme, commencing early, within two weeks of either hospital discharge or confirmed diagnosis (BACPR, 2017). These programmes typically include education on increasing physical activity and reducing sedentary behaviours with a focus on increasing patient understanding of the benefits of physical activity and empowering the patient to make behavioural adjustments during their recovery. This should be complemented by education on risk factor management, medications and coping with psychological responses to the patient’s condition.

Typically, exercise during cardiac rehabilitation is aerobic, involves large muscle groups, and is tailored around the patient’s capacity. This usually includes walking and circuit training. Aerobic exercise is complemented with stretching and flexibility and resistance training. Clinicians seeking further guidance on exercise frequency, intensity, type and duration should consult reputable and recent cardiac rehabilitation guidelines, for example, the British Association for Cardiovascular Prevention and Rehabilitation (BACPR), Standards and Core Components for Cardiovascular Disease Prevention and Rehabilitation 2017 (3rd Edition) (BACPR, London, 2017) and European Heart Network, Cardiac and Stroke Rehabilitation a European Heart Network paper (EHN 2018).

Recognising the low-resource circumstances in low- and middle-income countries, and lack of access that might be a consequence of poverty, health literacy, remoteness and other factors, there is an important need for flexible modes of delivery of cardiac rehabilitation and the provision of education and support in regard to physical activity and exercise. This may include telephone advice, online delivery and community-based education by medical or allied health professionals (Gallagher et al., 2017).

## Physical activity – other health benefits

### Physical Activity and Other Ncds

Physical activity is one of the most important ways to prevent and manage a wide range of NCDs. Adults who meet physical activity guidelines have a 20–30% reduced risk of all-cause premature death (WHO 2020 (1)). Adults who meet physical activity guidelines have a significantly lower risk of developing type 2 diabetes than do inactive adults (USDHHS 2018).

Physical activity is also protective against incident site-specific cancers of the breast, colon, bladder, as well as endometrial, oesophageal adenocarcinoma, gastric, and renal cancers (McTiernan et al., 2019; WHO 2020). Furthermore, high levels of physical activity could decrease the risk of multimorbidity, particularly cardiometabolic multimorbidity (He et al., 2021).

### PHYSICAL ACTIVITY AND Covid-19

There were adverse impacts on physical activity from the COVID-19 pandemic. Declines in physical activity occurred due to the combination of infection control practices such as closing common places for physical activity during the early days of the pandemic and inadequate efforts to promote physical activity. A review of 64 studies showed consistent decreases in physical activity and consistent increases in sedentary behaviour among both adults and youth during the pandemic (Stockwell et al., 2021). A meta-analysis of 16 studies of pre-pandemic physical activity among adult COVID-19 patients found a 43% lower risk of death among those who were active (Ezzatvar et al., 2022).

Experience during the COVID-19 pandemic demonstrated that this decline in physical activity had adverse consequences. Recent studies show that PA is associated with a strong immune response, risk reduction from community-acquired infectious disease and mortality, and increased vaccine potency (Nieman et al., 2019; Hamer et al., 2020) As physical activity was known to improve functioning of the immune system and reduce inflammatory processes (Chastin et al., 2021), WHO recommended physical activity during the pandemic (WHO, 2020). However, most governments did not promote physical activity. About 95% of COVID-19 hospitalizations were among people with inactivity-related NCDs, such as cardiovascular disease (Kompaniyets et al., 2021), which are more common among economically-disadvantaged groups.

The COVID-19 pandemic highlighted the importance of cardiovascular risk factors such as hypertension, obesity, smoking, type 2 diabetes and physical inactivity in patient outcomes. Physical activity was critical in helping people manage stress and maintain physical health through times of lockdowns and movement constraints. The pandemic also highlighted inequities in access to physical activity programmes, active neighbourhoods, walking and cycling facilities and open spaces.

Emerging from the COVID-19 pandemic, it is important to return physical activity levels back to pre-COVID levels, and then aim to increase them further. While further research is needed, physical activity is likely to play an important role in managing long COVID.

### Physical activity and strength

Muscle-strengthening activities like lifting weights can help increase or maintain muscle mass and strength, and improve muscle function (Winett & Carpinelli 2001). Muscle mass is an important determinant of chronic diseases such as type 2 diabetes and osteoporosis, and increased muscle strength is associated with an improved metabolic profile, and a reduced risk or cardiovascular disease and premature mortality (USDHHS 2018). Muscle mass declines with age, and resistance or strength exercises help older adults maintain functional capacity, independence and quality of life, and a reduced risk of falls (USDHHS 2018).

### Physical activity and mental health

Evidence supports the role of physical activity in improving mental health (Schuch et al., 2016), and health-related quality of life (Alphonsus et al., 2019). Physical activity is helpful in reducing risk of developing anxiety and depression. Adults with higher levels of physical activity were at reduced odds of developing anxiety (Schuch et al., 2019).

Evidence examining physical activity and symptoms of anxiety and depression indicate that physical activity also reduces symptoms in those who have anxiety (Gordon et al., 2017: Gordon et al., 2018) and depression (Gordon et al., 2018; Perez-Lopez et al., 2017). While there is insufficient evidence to establish an exact dose-response relationship for the impact of physical activity on mental health and health-related quality of life outcomes, available evidence is from studies typically assessing physical activity interventions of 3 or more times weekly (WHO 2020).

Newer research indicates that physical activity has a small yet significant effect on physical, mental and social domains of health-related quality of life (including symptoms of fatigue and depressive symptoms) (USDHHS 2018(2); Alphonsus et al., 2019).

### Physical activity and sleep

Moderate-to-vigorous physical activity improves the quality of sleep. Physical activity reduces the amount of time it takes to go to sleep, can increase the time in deep sleep and reduce daytime sleepiness (USDHHS 2018). There is evidence that both acute bouts and regular physical activity improve sleep and health-related quality of life outcomes in adults (USDHHS 2018).

### Physical activity and brain health

Emerging evidence supports the role of physical activity in preventing cognitive decline due to vascular factors or due to Alzheimer’s disease in older adults, and promoting brain development in early life (Guure CB et al. 2017). These benefits have been reported across a variety of types of physical activity, including aerobic activity, walking, muscle-strengthening activity, and yoga (Northey et al. 2018). The US Physical Activity Guidelines Committee reported that greater amounts of moderate-to vigorous-intensity physical activity are associated with improvements in cognition (e.g. processing speed, memory, and executive function) (USDHHS 2018).

## Physical activity and air pollution

**Figure:**
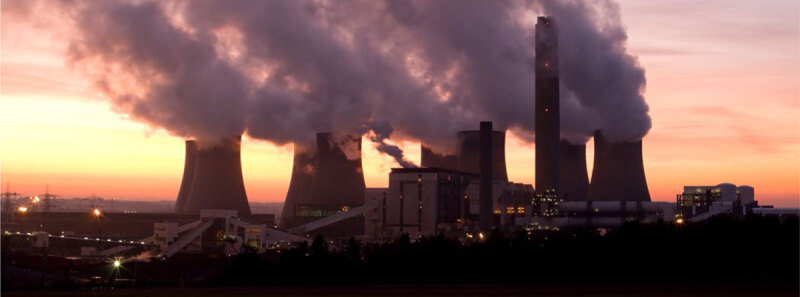


Outdoor and indoor air pollution, combined, are estimated to be responsible for over seven million premature deaths globally every year, making air pollution the fourth leading risk factor for all-cause mortality (Global Burden of Disease Risk Factor Collaborators, 2022). Cardiovascular causes are responsible for over 50% of this attributable mortality. The WHF has produced a policy brief on air pollution outlining health effects and recommendations to different sectors to reduce air pollution and provide advice to the general public and patients (WHF 2021).

While air pollution comes from many different sources, transport is considered to be one of the most significant in terms of detrimental health effects. Therefore, investments in policies and interventions that promote and support safe active transportation are essential for reducing fossil fuel consumption, air and noise pollution and reducing risk of cardiovascular disease (Macmillan & Woodcock 2017; WHO 2018).

Policy makers, city planners, the transport sector, and employers can all contribute to increasing the appeal, ease, incentive, and safety of travel by foot, by bicycle, or by public transport through the means outlined in Tables 7, 10. Less demand for car use can also reduce road expenditure and contribute to increased liveability in neighbourhoods, cities, and towns.

Short car trips are a substantial proportion of transport. In Austria over 40% of car trips are less than five kilometers (Katsis, Papageorgiou & Ntziachristos, 2014). In Germany the average car trip is less than 17 kilometers (German Federal Ministry of Transport and Digital Infrastructure, 2017). With appropriate policy, environmental supports and incentives many short car trips could be replaced by active modes, improving physical activity and reducing air pollution.

Does exercising in air pollution (e.g. cycling in traffic) offset the benefits of exercise, due to an increased inhalation of air pollution from greater respiration? While acute cardiovascular parameters are blunted to some degree by walking along a street with high traffic density compared to that of a park (Sinharay et al. 2018), the benefits of regular exercise far outweigh the detrimental effects of the somewhat higher dose of inhaled pollution. It has been estimated that a person would have to cycle for over 1 h 30 per day or walk for 10 h per day in levels of air pollution that exceed the most polluted megacities of the world (>100 µg/m^3^ PM2.5) before the benefits of exercise are lost (by comparison European urban centres typically fall in the range of 5–20 ~µg/m^3^ PM2.5; global urban average = 22 µg/m^3^) (Tainio et al. 2016). Additionally, air pollutants within vehicles can build up to be several times higher than the outside air under certain conditions, with motorists being exposed to higher levels of air pollution than cyclists with similar commutes (Karanasiou et al. 2014).

The elderly and individuals with cardiorespiratory diseases are groups that are particularly susceptible to the risks of air pollution. Guidance is available for physicians to advise patients on exercise in relation to air pollution (Giorgini et al. 2016) and various countries have developed online tools that show daily air quality together with broad health advice for the given air quality category, which often considers exercise (e.g. US Environmental Protection Agency: https://www.airnow.gov). Locations with high concentration of air pollution are often also underserviced and socioeconomically disadvantaged areas. Notwithstanding this, active transport can provide substantial net health benefits, irrespective of geographical context and the benefits from physical activity strongly outweigh any detrimental effects of traffic accident-related injuries and air pollution exposure (Mueller et al., 2015; Dinu et al., 2019).

## The economic cost of physical inactivity and benefit of physical activity

The health and economic cost of physical inactivity is considerable. According to the WHO, if there is no change in the current prevalence of physical inactivity, almost 500 million new cases of preventable NCDs will occur between 2020 and 2030 incurring treatment costs of just over US$300 billion or around US$ 27 billion annually.

Three quarters of this burden will be in low- and upper-middle-income countries (Santos et al., 2022: WHO 2022). These estimates are conservative as they do not include the cost of other important health outcomes such as preventable falls and related injuries in the elderly. Current models also do not include productivity losses due to morbidity and mortality (Hafner et al., 2020).

The good news is that physical activity is accessible to almost everyone and can have little or no cost. Effective interventions in settings such as primary care, schools, community settings and workplaces are cost effective (Zubala et al., 2017). There is a need to further methods and tools in studies to include the total health, social and economic costs and returns of increasing physical activity (WHO 2022).

## Physical activity and sedentary behaviour guidelines and benefits for sub-populations

In the last decade numerous countries as well as the WHO have produced physical activity guidelines.These have significantly advanced the field in several ways. Rigorous guideline processes have ensured systematic reviews were undertaken to update the evidence regarding the health benefits of physical activity as well as evidence regarding the dose (frequency, intensity and time of physical activity) required to achieve those health benefits.

Guidelines have also included in their terms broader and more contemporary issues such as the role of physical activity in mental, social and cognitive health, and the role of strength and flexibility in conferring additional or different health benefits. Importantly, the guidelines documents in some countries have also recommended evidence-based physical activity interventions and have informed polices regarding the ways in which population physical activity levels can be increased.

While we can be confident that humans share similar physiology, and the guidelines apply to us all, there are important differences when it comes to applying the guidelines in programmes at country and regional levels. This includes socio-economic, cultural, ethnic, climatic, religious, gender and social differences that impact on opportunities to achieve the guidelines. These factors need also to be prominent considerations in tailoring the way in which the guidelines are applied through appropriate national polices, action plans and programmes, as well as in public education, messaging and campaigns.

The WHO identified six key messages from its 2020 Guidelines on Physical Activity and Sedentary Behaviour

1. Physical activity is good for hearts, bodies and minds.
2. Any amount of physical activity is better than none, and more is better.
3. All physical activity counts.
4. Muscle strengthening benefits everyone.
5. Too much sedentary behaviour can be unhealthy.
6. Everyone can benefit from increasing physical activity and reducing sedentary behaviour.

**Figure:**
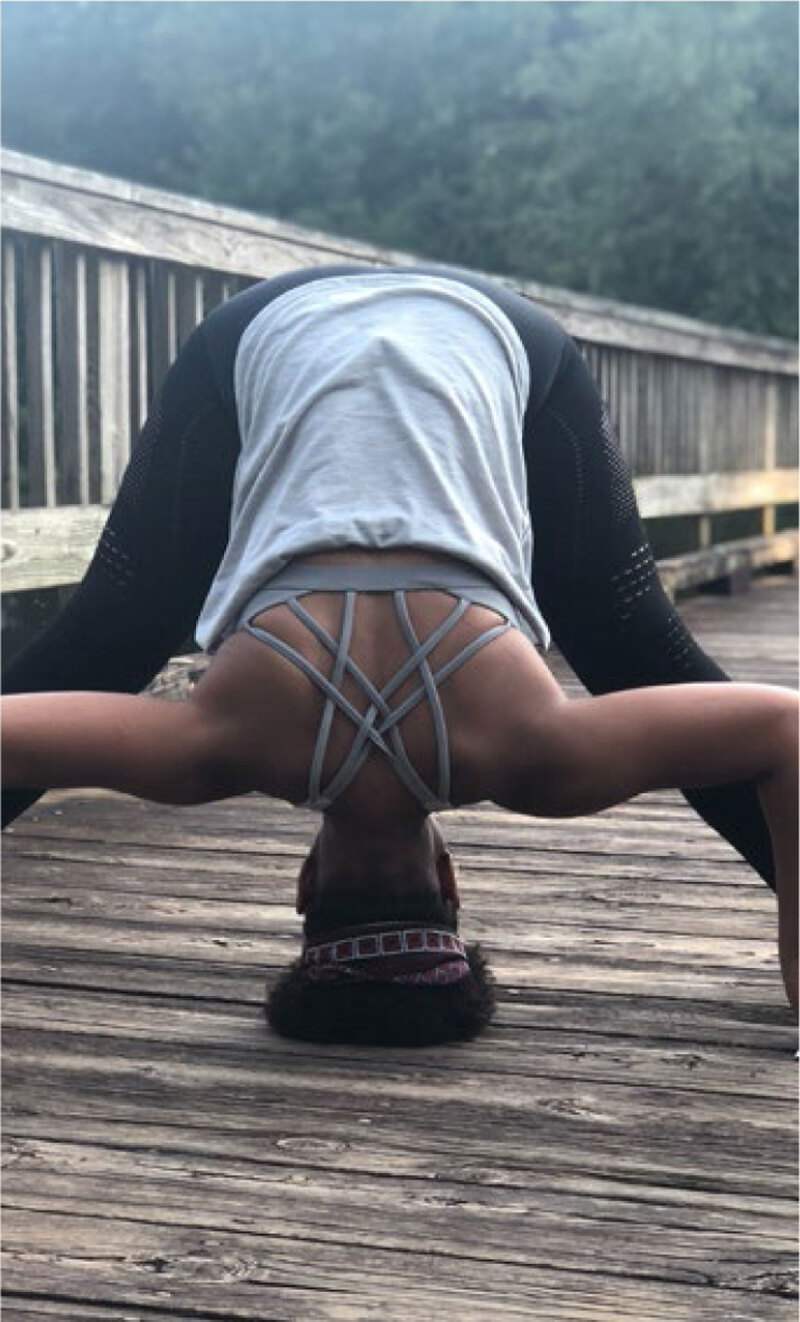

Guidelines have also been developed across the life-span, and across a broader range of population groups, enabling appropriate differences to be communicated for the early years, childhood, adolescence, adulthood and the senior years, as well as pregnant and post-partum women, and people living with disability. *These are outlined below*.

### Early years 0–5 years

**Benefits:** The first five years of life are fundamentally important to children’s growth and development. Active and energetic play is important for physiological, psychological and social developments in babies, infants and toddlers. Participation in physical activity is associated with improved motor and cognitive development, psychosocial and cardiometabolic health, physical fitness, bone and skeletal health, and adiposity (body fat) (Carson et al., 2017; Barnett et al., 2016; Leppänen et al., 2017; Saldanha-Gomes et al., 2017).

**Guidelines:** See Table 1.

**Table 1:** Physical activity and sedentary behaviour guidelines for the early years 0–5 years.


AGE	PHYSICAL ACTIVITY	SEDENTARY SCREEN TIME	QUALITY SLEEP

In a 24-hour day, **infants less than 1** year old should… 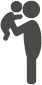	**Be physically active several times a day in a variety of ways**, particularly through interactive floor-based play; more is better. For those not yet mobile, this includes **at least 30 minutes in prone position** (‘tummy time’) spread throughout the day while awake.	**Not be restrained for more than 1 hour at a time** (e.g., prams/strollers, high chairs, or strapped on a caregiver’s back); Screen time is not recommended. When sedentary, engaging in reading and storytelling with a caregiver is encouraged.	**Have 14–17 hours** (0–3 months of age) **or 12–16 hours** (4–11 months of age) **of good quality sleep**, including naps.

In a 24-hour day **children 1–2 years of age** should… 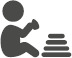	**Be physically active several times a day in a variety of ways**, particularly through interactive floor-based play; more is better. For those not yet mobile, this includes **at least 30 minutes in prone position** (‘tummy time’) spread throughout the day while awake.	**Not be restrained for more than 1 hour at a time** (e.g., prams/ strollers, high chairs, or strapped on a caregiver’s back) or sit for extended periods of time. **For 1 year olds, sedentary screen time (such as watching TV or videos, playing computer games) is not recommended**. **For those aged 2 years, sedentary screen time should be no more than 1 hour; less is better**. When sedentary, engaging in reading and storytelling with a caregiver is encouraged	**Have 11–14 hours of good quality sleep**, including naps, with regular sleep and wake-up times.

In a 24-hour day, **children aged 3–4 years of age** should… 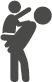	**Spend at least 180 minutes in a variety of types of physical activities** at any intensity, of which at least 60 minutes is moderate- to vigorous-intensity physical activity, spread throughout the day; more is better.	**Not be restrained for more than 1 hour at a time** (e.g., prams/strollers) or sit for extended periods of time. **Sedentary screen time should be no more than 1 hour; less is better**. When sedentary, engaging in reading and storytelling with a caregiver is encouraged.	**Have 10–13 hours of good quality sleep**, which may include a nap, with regular sleep and wake-up times.


### Children and adolescents aged 5–17 years

**Benefits:** Physical activity is important for healthy growth and development in children and young people. Children who meet or exceed physical activity guidelines experience improved physical, social, psychological and cognitive (thinking and mental skills) health. Higher levels of physical activity are associated with additional health benefits.

Participation in physical activity is associated with reduced adiposity; improved cardiometabolic health; improved physical fitness and bone health; motor skill development; quality of life; and reduced psychological distress (Poitras et al., 2016); prosocial behaviour (Singh et al., 2019); improved sleep duration and quality (Belmon et al., 2019); and improved mental health (Stanczykiewicz et al., 2019).

**Guidelines:** See Table 2.

**Table 2:** Physical activity and sedentary behaviour guidelines for children and adolescents aged 5–17 years.


AGE	PHYSICAL ACTIVITY

**Children and adolescents** aged 5–17 years 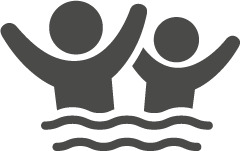	- **60 minutes per day** – of moderate to vigorous-intensity, mostly aerobic, physical activity, across the week. - **At least 3 days a week** – of vigorous-intensity aerobic activities, as well as those that strengthen muscle and bone, should be incorporated at least 3 days a week. - **Limit** the amount of time spent being sedentary, particularly the amount of recreational screen time.


### Adults aged 18–64 years

**Benefits:** Moderate-to-vigorous physical activity (MVPA) decreases the likelihood of adults developing and suffering from coronary artery disease. Light physical activity (LPA) is also associated with health outcomes including reduction in all-cause mortality. Physical activity can also favourably impact other modifiable risk factors including reductions in high blood pressure, helping to maintain a healthy weight, and primary prevention of type 2 diabetes.

Physical activity is also protective against incident site specific cancers. Physical activity can improve mental health, is helpful in both the prevention and treatment of depression and anxiety and can help reduce social isolation, increase social support, and strengthen social connections in communities.

**GUIDELINES:** See Table 3.

**Table 3:** Physical activity and sedentary behaviour guidelines for adults aged 18–64 years.


AGE	PHYSICAL ACTIVITY

**Adults** aged 18–64 years 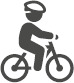	- **At least 150–300 minutes of moderate-intensity aerobic physical activity, per week**. **or at least 75–150 minutes of vigorous intensity aerobic physical activity**, per week; **or an equivalent combination** of moderate- and vigorous-intensity activity throughout the week, for substantial health benefits. **On 2 or more days a week Adults should do muscle strengthening activities** at moderate or greater intensity that involve all major muscle groups, as these provide additional health benefits. - **For additional health benefits, increase moderate-intensity aerobic physical activity to more than 300 minutes;** or do more than 150 minutes of vigorous-intensity aerobic physical activity; or an equivalent combination of moderate- and vigorous-intensity activity throughout the week. This is also important to help reduce the detrimental health effects of high levels of sedentary behaviour. - **Limit the amount of time spent being sedentary**. - **Replacing sedentary time with physical activity of any intensity (including light intensity) provides health benefits**.


### Older adults aged 65 and older

**Benefits:** Physical activity is critical to maintaining people’s quality of life as they age. Being active is strongly associated with healthier ageing (Daskalopoulou et al., 2019). Studies conducted in older adults without pre-existing cardiovascular disease demonstrate that physical activity significantly reduces mortality risk from heart disease (Shiroma et al., 2010). Muscle-strengthening activities like lifting weights can help older adults increase or maintain muscle mass and strength, functional capacity, independence and quality of life. This is important for older adults who experience reduced muscle mass and muscle strength with aging. Finally, emerging evidence supports the role of physical activity in preventing cognitive decline and Alzheimer’s disease in older adults.

Initiation of physical activity in later adulthood also lowers the risk of cardiovascular disease. It is never too late to start! So, encouraging people to become active or to keep up their physical activity as they age is an important element of primary care and aged care. It supports community interaction and facilitates social engagement.

**Guidelines:** See Table 4.

**Table 4:** Physical activity and sedentary behaviour guidelines for older adults aged 65 years and older.


AGE	PHYSICAL ACTIVITY

**Older Adults** aged 65 years and older 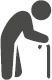	- **At least 150–300 minutes of moderate-intensity aerobic physical activity, per week;** **◦ or at least 75–150 minutes of vigorous intensity aerobic physical activity;** **◦ or an equivalent combination** of moderate- and vigorous-intensity activity throughout the week, for substantial health benefits. - **On 2 or more days a week do muscle strengthening activities** at moderate or greater intensity that involve all major muscle groups, as these provide additional health benefits. - **Increase moderate intensity aerobic physical activity to more than 300 minutes;** or do more than 150 minutes of vigorous-intensity aerobic physical activity; or an equivalent combination of moderate- and vigorous intensity activity throughout the week, for additional health benefits. - **Limit the amount of time spent being sedentary**. Replacing sedentary time with physical activity of any intensity (including light intensity) provides health benefits. - **Aim to do more than the recommended levels of moderate- to vigorous intensity physical activity** to help reduce the detrimental effects of high levels of sedentary behaviour on health.


### Pregnant and post-partum women

**Benefits:** Physical activity during pregnancy and postpartum is associated with decreased risk of pre-eclampsia, gestational hypertension, gestational diabetes, excessive gestational weight gain, delivery complications and postpartum depression. Physical activity can be incorporated into daily routines both during and after pregnancy (Table 5).

**Guidelines:**

**Table 5:** Physical activity and sedentary behaviour guidelines for pregnant and post-partum women.


AGE	PHYSICAL ACTIVITY

**Pregnant and post-partum women** 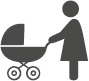	- **Undertake regular physical activity throughout pregnancy and postpartum**. - **In addition:** Women who, before pregnancy, habitually engaged in vigorous intensity aerobic activity, or who were physically active, can continue these activities during pregnancy and the postpartum period. - **Do at least 150 minutes of moderate intensity aerobic physical activity throughout the week for substantial health benefits**. - **Incorporate a variety of aerobic and muscle strengthening activities**. - **Adding gentle stretching may also be beneficial**. - **Limit the amount of time spent being sedentary**. - **Replacing sedentary time with physical activity of any intensity (including light intensity)** provides health benefits.


### Children and adults living with disabilities

**Benefits:** People with disabilities are less likely than other groups to meet physical activity guidelines (Table 6). However, meta-analyses demonstrate physical activity has beneficial effects on cardiovascular fitness, musculoskeletal fitness, cardiometablolic risk factors, brain health, and mental health outcomes. There is evidence physical activity can contribute to the prevention and management of coronary heart disease, type 2 diabetes, stroke and some types of cancer among people with disabilities. (Carroll DD et al., 2014: Ballard-Barbash 2012).

**Guidelines:**

**Table 6:** Physical activity and sedentary behaviour guidelines for people living with disabilities.


AGE	PHYSICAL ACTIVITY

**People with disabilities** 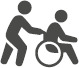	**There is no evidence to suggest that children and adults with disabilities should not aim to achieve the same levels of physical activity as other people of the same age**. Therefore, both the children and adolescents and the adult guidelines are also applicable to people living with a disability. However, people living with disability may need to consult a health-care professional or other physical activity and disability specialist to help determine the type and amount of activity appropriate for them.


## Global Physical activity Prevalence

The high prevalence of physical inactivity is an important contributor to the overall burden of physical inactivity. *These are outlined below*.

**Figure:**
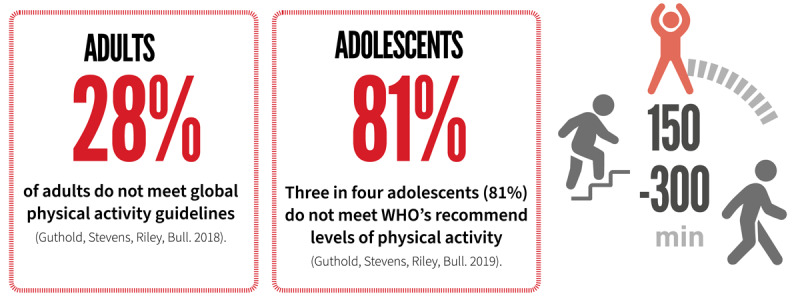

There is yet no comparable data on global physical activity prevalence for younger children.

Physical activity prevalence is extensively addressed in the WHO Global status report on physical activity, released in October 2022 (WHO 2022). Striking findings from this world-first report indicate physical activity rates vary across nations and sub-population groups. There are inequalities in physical activity between many sub-groups in the population including:

- **Women are less active than men**.
- **Girls are less active than boys**.
- **Older adults are less active than younger adults**.
- **Those living in socioeconomic disadvantage are less active than the advantaged**.
- **People with disabilities are less active than the broader community**.

## Health policy connections for physical activity

### WHO Global Action Plan for the Prevention and Control of Noncommunicable Diseases (NCDs) (2013)

Physical activity was identified as a policy priority in the WHO Global Action Plan on Noncommunicable Diseases (GAP NCD) (WHO, 2013). Priority policy actions were identified that would help member states achieve the target identified in the accompanying WHO NCD Monitoring Framework, to *‘reduce global physical inactivity by 10%’*. This was subsequently updated to the global target of a 15% relative reduction in population levels of physical inactivity by 2030.

In September 2018, the United Nations General Assembly held its third High-Level Meeting on the prevention and control of NCDs. Following a comprehensive review of the global and national evidence, as well as progress achieved, the Political Declaration (United Nations 2018) included a fifth disease condition, mental health, and a fifth risk factor, air pollution. These both have important relevance to cardiovascular health and to physical activity.

The WHO objectives for noncommunicable disease prevention and control now incorporate recommended actions for five disease conditions and five risk factors.

**Disease conditions:** Cardiovascular disease, Diabetes, Cancers, Chronic respiratory disease, mental health

**Risk factors:** Promoting physical activity, promoting healthy diet, tobacco control, reducing the harmful use of alcohol, reducing air pollution of studies were conducted to understand the “abuse potential” or “abuse liability” of NRTs. Evidence suggests that the use of NRTs outside of therapeutic context is rare.

### WHO Global Action Plan on physical activity

In 2018, following an extensive global consultation process, the WHO released the **Global Action Plan on Physical Activity 2018–2030 – More Active People for a Healthier World (WHO GAPPA)** (WHO 2018).

The WHO GAPPA extensively outlines a vision for a more active world and “…*provides updated guidance and framework for effective and feasible policy actions to increase physical activity at all levels”* (WHO 2018). The plan provides a framework for effective implementation of physical activity policies and action plans. It outlines four strategic policy areas – active societies, active environments, active people and active systems – and includes twenty policy actions within health and across sectors (WHO 2018).

### National physical activity action plans

The WHO GAPPA was adopted by resolution at the World Health Assembly in 2018. As a result, member states have committed to develop national physical activity action plans.

A number of countries have done so; however, implementation has been slow and the *WHO Global Status Report on Physical Activity 2022* reports that only two WHO GAPPA policy indicators show implementation by over three-quarters of countries (WHO 2022). This highlights the urgency for increased advocacy to accelerate commitments to implementation of national physical activity action plans, based on WHO GAPPA, in all countries.

## Non-health policies in multiple sectors help determine physical activity levels

While the primary focus of this WHF policy brief is the cardiovascular and other health benefits of physical activity, it is important to recognise the considerable co-benefits of physical activity in non-health domains (Munzel et al., 2021).

The cross-sectoral nature of physical activity highlights the critical importance of partnerships, between health professionals and professionals from transport, education, planning, sport, education and local government to achieve both health benefits and cross-sector co-benefits through initiatives that enable physical activity. Policy changes in many sectors are needed to create long-term improvements in physical activity (Giles-Corti et al., 2022). The policy intersects are at multiple levels, from national to sub-national as well as local administrations, governments and municipalities.

Often the same interventions that increase physical activity and active transport can deliver a range of co-benefits including reduced air pollution, reduced traffic congestion, increased sport sector participation/engagement, enhanced social support, stimulus to economic activity, increased social capital and more equitable access to destinations such as retail, community services and facilities and places of worship. Many of these settings are also critical locations for physical activity programmes and for the delivery of educational messages.

Relevant policies and legislation regarding land use (zoning), transportation and parks can be made at the national, regional (e.g., state), and local levels and have important impacts in health and more broadly. Healthy and active neighbourhood design contributes to increased physical activity as well as social safety and inclusion, and increased social capital from more convivial streets, spaces and places (Sallis et al., 2015).

Similarly, policies in education, sport, recreation and parks are typically not designed with physical activity and health in mind, but voluminous research shows a wide variety of policies can affect physical activity (Giles-Corti et al., 2016; Young et al., 2020). Policies also affect the equity of distribution of physical activity-relevant built environment features both across cities/towns and across neighbourhoods within cities/towns (Lowe et al., 2022).

This situation presents opportunities for strategic collaboration that extend the limited resources of physical activity and health advocates. There are already robust advocacy groups in many countries related to air pollution, climate change, smart growth (walkable communities), walking and bicycling, public transport, public open space, injury prevention, economic development, and health equity. Because designing environments to support physical activity is consistent with achieving all these desirable outcomes (Sallis et al., 2015), physical activity advocates can (and should) magnify their impact by partnering with an array of existing groups to achieve common goals that will achieve co-benefits (United Nations, 2015).

It is important to assess environmental equity in each locality and to target interventions to those most in need (Boeing et al., 2022). Governments should prioritise providing safe and accessible walking, cycling, and public transport infrastructure and safe, accessible outdoor spaces in low-income neighbourhoods. Fiscal incentives such as subsidising the cost of public transport or bicycles and sport participation can reduce inequity and drive demand for physical activity and active modes of transport. Moreover, there is an urgent need to benchmark and monitor progress to ensure that cities, towns and communities are transitioning in healthy and sustainable ways (Giles-Corti et al., 2022).

**Figure:**
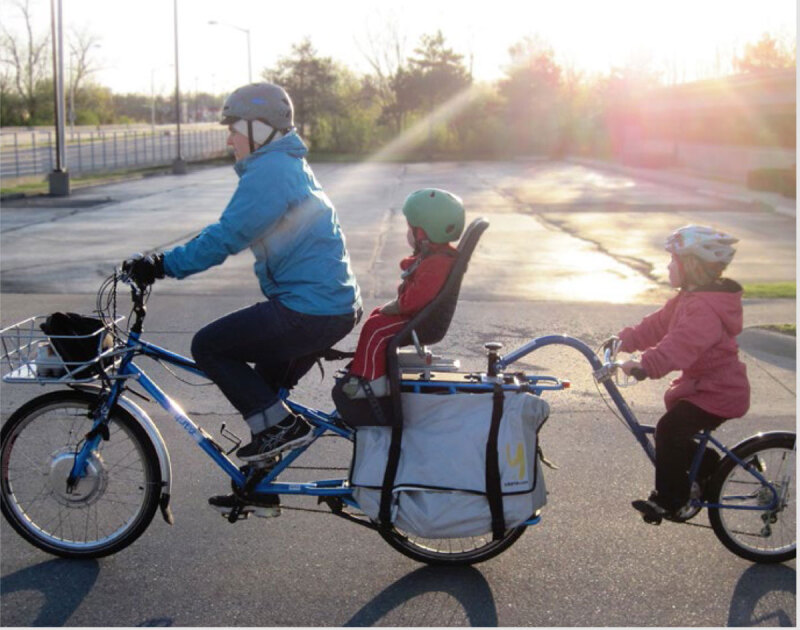

**Case study: Australia**

**Healthy Active by Design**

The National Heart Foundation of Australia has developed online design guidance for urban planners.

The website healthyactivebydesign.com.au was created to highlight how best-practice planning and design of buildings, streets, towns and cities can improve health. The practical guide offers evidence, advice and examples to assist with the development of healthy and active spaces and places.

Healthy Active by Design is organised around eight design features:

- Public open space
- Community facilities
- Buildings
- Destinations
- Movement networks
- Housing diversity
- Sense of place, and
- Healthy food

Visit www.healthyactivebydesign.com.au

**Figure:**
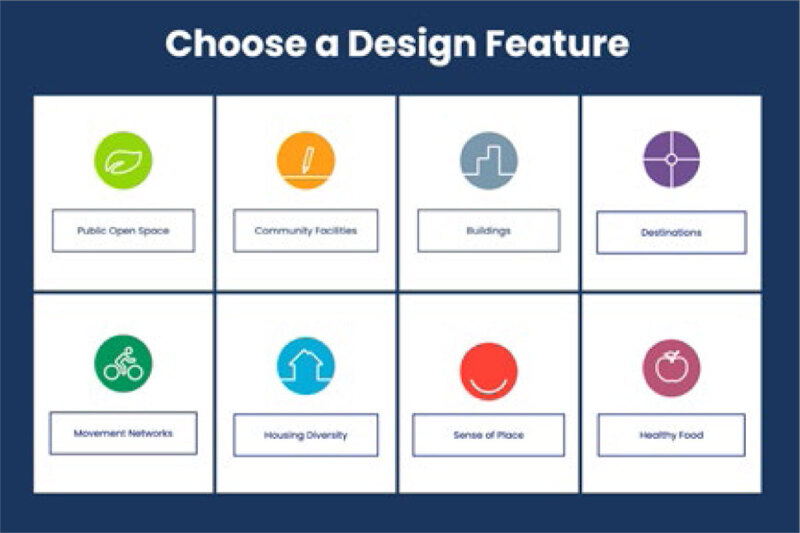

**Figure:**
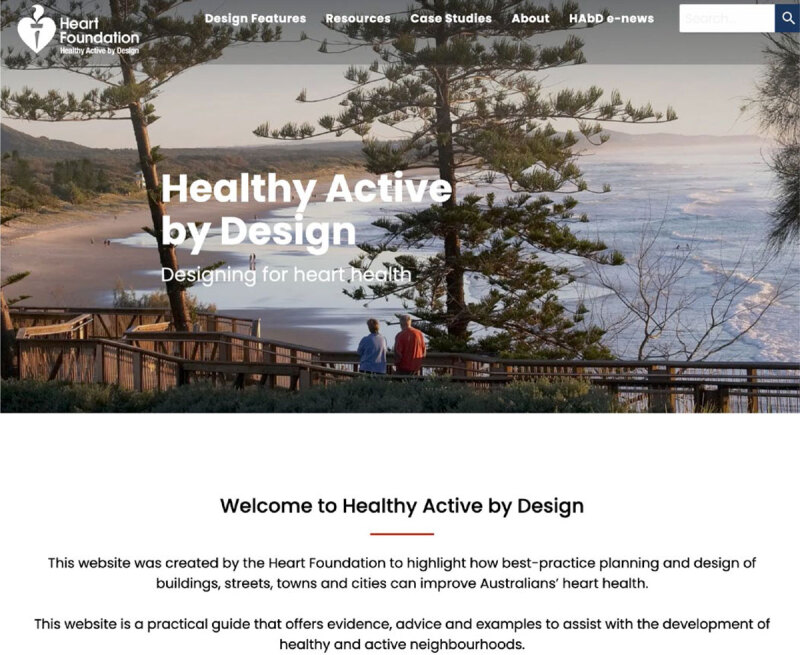


## World Heart Federation Physical Activity Recommendations

### Effective Interventions for Increasing Population Levels of Physical Activity

The rapid advance of evidence establishing the cardiovascular health benefits of physical activity has been vitally important in positioning physical activity as a global health policy priority for the UN and the WHO.

Of equal importance is the expanding evidence base to inform the implementation of effective community, public policy and systems-level interventions. Governments, policy makers, communities and professionals require guidance to inform polices and interventions that are most likely to increase physical activity, across the population as well as for population sub-groups, and promote equity.

Uptake and dissemination of these effective (evidence-based) interventions has been slow, and as with other areas of NCD prevention, implementation of effective policies and interventions has lagged behind the evidence (Horton, 2016; United Nations, 2018 (2), WHO 2022).

### The World Heart Federation Supports the Who Global Action Plan on Physical Activity 2018–2030

The WHO GAPPA provides a framework for the selection of appropriate policy and effective actions for local advancement of physical activity, to increase physical activity for all people. The WHF supports GAPPA and its four strategic policy areas – active societies, active environments, active people and active systems; as well as the accompanying twenty policy actions within health and across sectors. The WHO GAPPA, combined with the *ISPAH 8 Investments that Work*, provide a menu for effective investments for physical activity and for global and local action.

**Figure:**
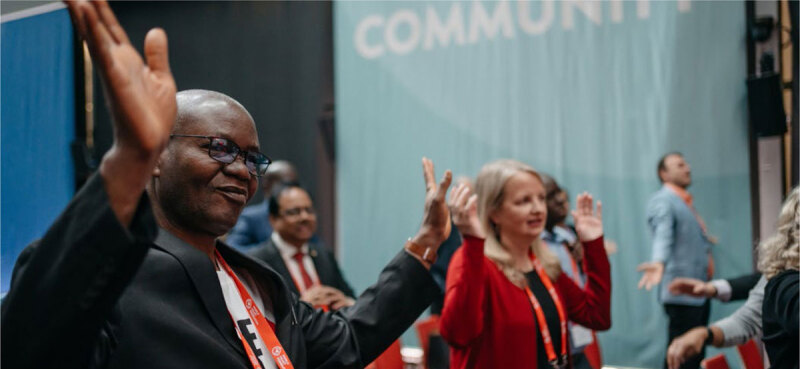


### Overarching World Heart Federation policy recommendation

All nations develop and implement a comprehensive National Physical Activity Policy, with implementation supported by a funded National Action Plan.

**Figure:**
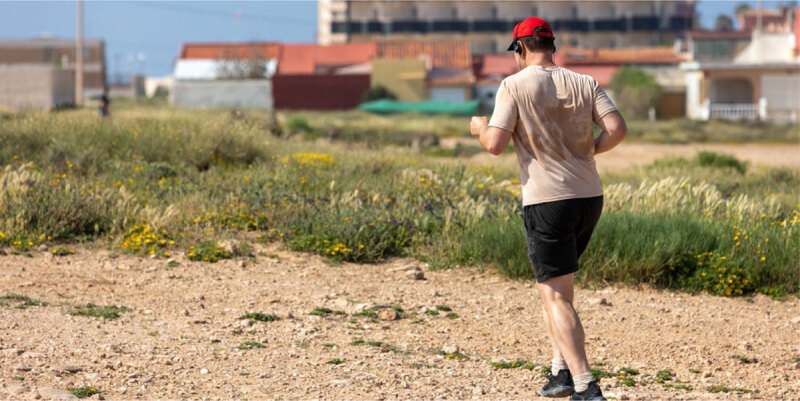


### Supporting recommendations

Tables 7 to 10 illustrate a comprehensive range of policy and practice recommendations and actions for physical activity, based on evidence of effectiveness. These are organised around the WHO GAPPA four strategic policy areas. The Tables include the WHF’s recommendations on measures to increase physical activity, amalgamating advice and evidence from various sources.

**Local context:** To maximise benefits of these physical activity initiatives it is important to consider community needs, culture, geography and the social and economic determinants of inactivity. Policies and programs should be adapted to take account of community needs and local evidence. This is relevant in all countries, but particularly in low-resource circumstances in low –and middle-income countries, and in environments where poverty, health literacy, remoteness and other factors may otherwise impact effectives or reduce access.

**Figure:**
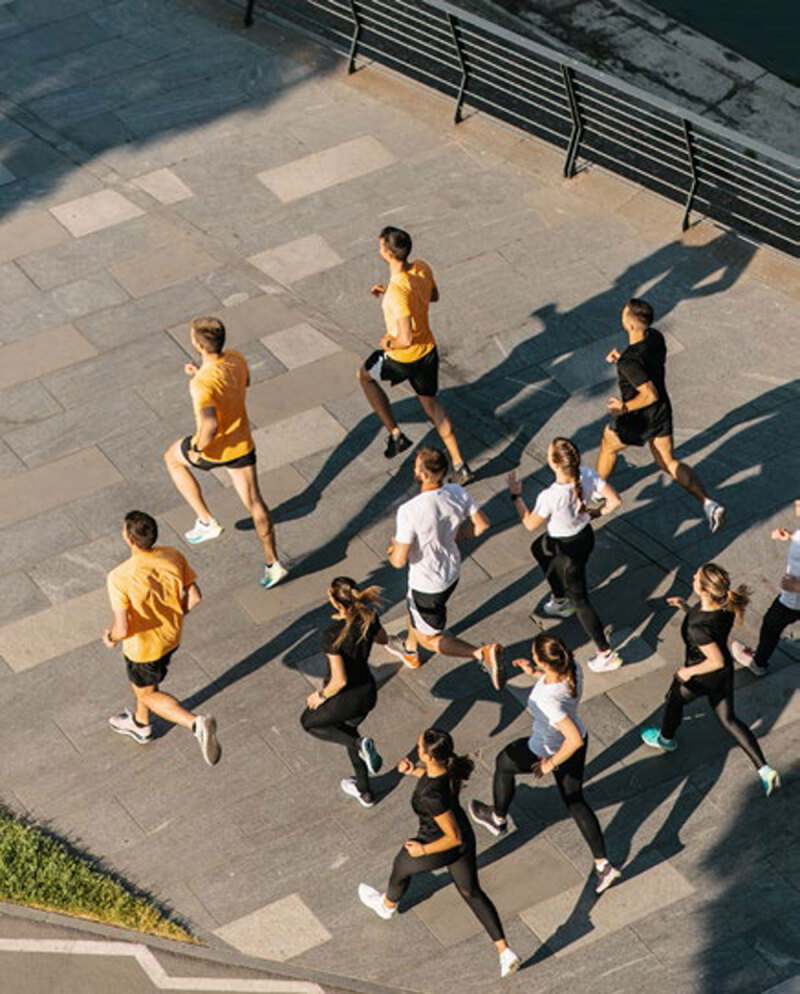

**WHO Strategic objective**

**CREATE ACTIVE SOCIETIES – Social norms and attitudes**

This includes policy actions and interventions that build knowledge and understanding of physical activity and its many benefits as well as how to access physical activity in your community context in a way that best meets needs. These actions over time will also contribute to changing community norms about physical activity.

**Table 7:** Recommended actions to create active societies.


RECOMMENDED ACTIONS TO CREATE ACTIVE SOCIETIES	WHAT WORKS?	KEY ACTORS

**Communications and media: Implement communications, public education and mass media campaigns**. This includes paid media, social media, and free-to-air media generated through public relations and advocacy	Large scale national mass media can be effective in setting a community agenda around physical activity, and are recommended as a ‘best buy’ for NCD prevention (WHO 2017). Mass media can be effective in raising awareness, knowledge and intention for physical activity (Stead et al., 2019). Media that is based on sound psychological theory and social marketing principles (Williamson et al., 2020). New digital and online forms of media are cost effective and nimble in reaching a defined audience and responding to an evolving agenda (Bergeron CD et al., 2019). Messages, images and delivery modes that are thoroughly market-tested and tailored to the intended audience (Williamson et al., 2020). Unpaid media and public relations is important for raising public discussion about physical activity.	**Governments at national and sub-national levels** **Communications specialists** **Foundations**

**Mass participation events: Implement regular mass participation initiatives**	Mass-participation events that are made accessible being provided free and which can effectively engage large numbers of individuals, groups and families in being active in a social setting. Mass events have been effective in increasing walking and cycling. This includes the Ciclovia programme, implemented successfully to mass audiences in complex urban settings such as Bogota (Torres et al., 2013). Events such as car-free-days can attract large participation numbers while also setting an advocacy agenda around active mobility and attracting media attention (see case study).	**Governments at city and local levels** **Civil society**

**Training: Strengthen pre- and in-service training of professionals, within and outside the health sector**	Strengthening on-the job training of primary care workers and allied health professionals to incorporate the benefits of physical activity, and how to deliver effective interventions and/or referral to community programmes and settings.	**Universities** **Professional societies**

**WHO Strategic objective**

**CREATE ACTIVE SOCIETIES – Spaces and Places**

This includes policy actions that strengthen design guidance to enable increased physical activity, walking and biking and equity and accessibility. This includes reforms in urban planning and transport systems, nature and in and around settings such as buildings, schools, workplaces, health care, public housing and sports facilities.

**Table 8:** Recommended actions to create active environments.


RECOMMENDED ACTIONS TO CREATE ACTIVE ENVIRONMENTS	WHAT WORKS?	KEY ACTORS

**Walking, rolling and cycling network infrastructure: Ensure provision of walking, rolling and cycling infrastructure to enable and incentivise greater physical activity and access by walking, cycling, and mobility-assist devices**	**It is important to take account of the needs of LMICs when assessing suitability of interventions**. Road safety is an important consideration for mixed zoning in LMIC communities. Congestion is an important contributor to road crashes and resulting deaths and injuries. Walking and cycling safety need to be primary considerations when planning for commercial and mixed zones. Active transportation policies operate at three levels to provide for a comprehensive approach: **Macro-scale – City planning, land use policy, urban and transport planning**. Where decision makers create and enable environments to ensure access to destinations required for daily living by walking or bicycling, and mixed-use zoning with a diversity of destinations thereby reducing vehicle dependency. **Medium (meso) scale – Pedestrian and bicycle networks, and infrastructure such as Complete Streets policies and Safe Routes to School initiatives**. Where city planners need to create networks of facilities and infrastructure that provide safe and attractive places for walking and bicycling, ensure high-quality and high-frequency public transit, and ensure there is safe cycling infrastructure within 5km of public transit, shops, services and schools.	**Governments at national, sub-national and local levels** **Transport authorities** **Transport and city planning professionals and societies** **Health professionals**

	**Micro-scale – Local design interventions and place-making such as building orientation and access, street furnishings, and safety and traffic calming measures**. Where local elements of communities are designed to support physical activity such as streetscapes with sidewalks/footpaths of adequate width, safe places to cross streets, trees or awnings to protect pedestrians from weather, curb ramps and other features for the benefit of people with disabilities and parents, traffic calming, speed reductions, protected space for bicycles, parks with facilities that support multiple types of physical activities and sports, and that appeal to people of all ages, and complementary programmes such as walk to school, or walk to work programmes, car-free days, bike hire/purchase schemes and public education. Low income should not be a barrier to active transport. Fiscal incentives such as subsidising the cost of public transport or bicycles can reduce inequity and drive demand for active modes of transport. Use of the WHO HEAT tool is recommended to support economic assessment of investment in walking and cycling networks and new infrastructure (WHO HEAT Tool).	

**Healthy urban planning policies: Prioritise compact, mixed-land use that integrates cities, towns and villages, including those in rural communities, with safe and accessible walking, cycling, public transport, sport, recreation, and public open space infrastructure**	Promote compact, mixed land use to create connected and walkable neighbourhoods and enable greater accessibility to schools, shops and key services. In LMIC countries ensure that mixed land use policies are accompanied by prioritisation of safety, comfort and desirability for walking and active transport are prioritised. Improve access to public transport, particularly for disadvantaged or vulnerable populations. Physical activity levels are higher in neighbourhoods with higher residential density, a more connected street network, a good public transport network, and more parks (Sallis et al., 2016). Diverse and affordable housing with high enough residential density to support many nearby shops and services and frequent public transit service. Connected street networks that allow direct routes to destinations. These design principles apply similarly in big cities and small towns, though they may not be directly applicable to truly rural and remote areas.	**Governments at national, sub-national and local levels** **Urban planning authorities** **Urban planning professionals and societies** **Health planners and health professionals** **Health professional societies**

**Public and green open spaces: Strengthen access to well-designed public open spaces, green spaces, play spaces, parks and nature, especially in LMIC settings**	Regulation that requires connected networks of green spaces and places that ensure equitable access. Nearby parks and public open space (see below) that have amenities such as shade trees, toilets, water fountains, and benches for resting whenever possible. Shopping areas, transit stops, schools, and workplaces should be designed to facilitate access by walking, bicycling, and public transport by providing end-of-trip facilities. Provision of safe and accessible green spaces in low-income areas which tend to have lower access to public open spaces (Astell-Burt et al., 2014).	**Governments at national, sub-national and local levels** **Urban planning authorities, professionals and societies** **Parks and gardens authorities and professionals** **Health professionals and societies**

**Road safety: Increase policy and environment actions to ensure safety for all walkers and cyclists** with a particular emphasis on vulnerable road users (children, the elderly and people with disabilities)	Policy actions that improve road safety and the personal safety of pedestrians, cyclists and others using mobility devices/aids with wheels (wheelchairs, scooters and skates) consistent with the WHO and UN Decade of Action on Road Safety (WHO and UN 2011). Reducing traffic volumes and speeds, and introduction of traffic calming measures, prioritizing local neighbourhoods where people walk and cycle to local services and settings. Ensuring bike lanes separated from road vehicles, and pedestrian crossings. Policy priority is given to environments and settings where actions can reduce risk for the most vulnerable populations (e.g. children, the elderly and people with disabilities). Partnerships with road safety planners and agencies. Teaching road safety skills to children of all ages, including in the school curriculum.	**Governments at national and local levels** **Transport and road safety professionals and societies** **Health, safety and injury prevention professionals and societies** **Police** **Schools/education**

**Reduce air pollution: Implement policy actions and strengthen infrastructure to minimize exposure to traffic related air pollution**	**Note synergy of actions with walking and cycling network infrastructure, and healthy urban planning policies**. Policy actions and investments in safe and green active travel alternatives to car travel. Ensuring well connected infrastructure to increase usability. Ensuring transport policy gives due consideration to minimizing any risk of increased exposure to traffic related air pollution, especially in locations with high concentration of air pollution. These areas are often also underserviced and socioeconomically disadvantaged areas. Providing disincentives (e.g. low-emission zones, vehicle and fuel taxing, parking charges) and incentives (e.g. bike-to-work schemes). Use of no-idling/no vehicle access at schools at start and end of the day. Communication campaigns highlighting the co-benefits of active travel (for health, air pollution and climate change).	**Governments at National and city levels** **Environment professionals** **Civil society organizations** **Health professionals and scientific societies**

**Case study: Nigeria**

**Car free day for active and inclusive transport in nigeria**

The Government of the State of Lagos in Nigeria recently (September 25, 2022) implemented the first ever ‘Car Free Day’ initiative in Nigeria to encourage the over 20 million population in the city to take up active and inclusive modes of transport such as cycling and walking (Lagos State Government, 2022). The initiative also aims to create awareness about the health and environmental benefits of non-motorized transport, including improved levels of physical activity and reduced environmental pollution. In a previous similar initiative, Lagos recorded a marked reduction in vehicular movement, improved air quality as well increased leisure time physical activity during the COVID-19 lockdowns (Lawanson et al., 2021). The imposed restriction on the use of motorized transport allowed many residents of all ages in the city to use the streets for multiple types of physical activity such as playing sport, running/jogging, walking, bicycling and other active play throughout the day. While the health impact of the ‘Car Free Day’ initiative is yet to be evaluated in Lagos, it clearly has the potential to reduce air pollution, road traffic accidents and congestion, and improve physical activity practices and inclusive mobility on a sustainable and population wide basis.

https://lagosstate.gov.ng/blog/2022/09/24/lagos-to-observe-car-free-day-on-september-25-2022/

https://transportation.lagosstate.gov.ng/2022/09/13/lagos-to-observe-car-free-day-on-september-25-2022/

**Figure:**
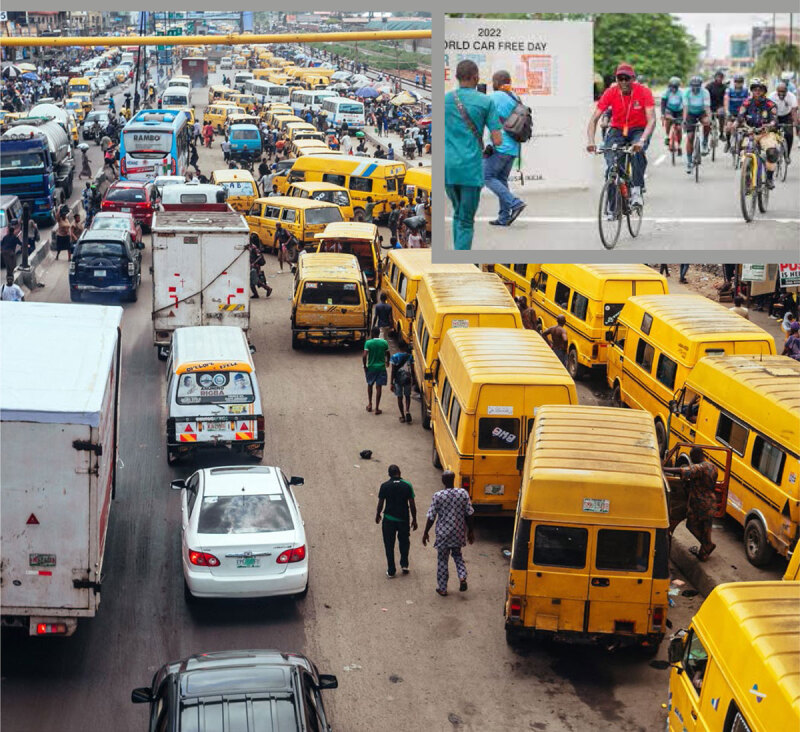

**WHO Strategic objective**

**CREATE ACTIVE PEOPLE – Programmes and opportunities**

This includes the provision of programmes and opportunities to be active across multiple settings. This promotes access and equity whereby people of all ages and abilities, families and communities can engage in regular physical activity.

**Table 9:** Recommended actions to create active people.


RECOMMENDED ACTIONS TO CREATE ACTIVE PEOPLE	WHAT WORKS?	KEY ACTORS

**Whole of school programmes: Implement multi-component approaches to provide physical activity opportunities within and beyond the school day**	Research has shown multi-strategy interventions, incorporating the whole school, to be effective in increasing physical activity policy implementation (Nathan et al., 2022) and therefore the amount of school-based physical activity delivered. Multi-strategy (or comprehensive) school physical activity programmes that include the following elements: Quality, effective physical education Supportive school facilities including play spaces Supportive environments for walking and cycling to school Programmes to support physical activity for all children School sports (recreational and competitive) Classroom physical activity breaks (U.S. CDC 2018). Policy areas with stronger evidence of physical activity impact include physical education lessons, school sport, classroom-based physical activity, active school breaks, and shared use agreements (Woods et al., 2021).	**Education authorities** **School principals and leaders** **Teachers, physical educators and their professional societies** **Health professionals** **Parents and carers**

	This demonstrates the need for, and importance of, school physical activity policy. This informs school-based practices and ethos around establishing a physically active school based on evidence and frameworks such as the Creating Active Schools Framework (Daly-Smith et al., 2020). Importantly, school-based interventions represent an opportunity for programme delivery at scale.	

**Active Healthcare: Support health care systems, hospitals and primary care to promote and implement physical activity policies and interventions for patients in primary care and those recovering from heart disease**	Establish standardized measures for physical activity assessment, prescription and referral in health care delivery (Smith, Marshall & Huang, 2004; Whitsel et al., 2020). Develop quality and performance measures for clinicians to assess, counsel and refer for physical activity prescription. Ensure remuneration/payment schemes are in place for assessment and referral, and for supervised exercise therapy. Provide physical activity brief intervention, counselling and referral as part of routine primary health care can motivate behaviour change (WHO 2018, HEARTS Technical Package, 2018). WHO recommends brief interventions on physical activity as a cost-effective “best buy” when it comes to the management and primary prevention of NCDs. Ensure patients recovering from myocardial infarction receive an individualised exercise and lifestyle assessment. This combined with consideration of diagnosis, risk factors, functional capability and participant preferences, better enables provision of a tailored |exercise programme of appropriate and increasing intensity, frequency and duration. Provision of exercise as part of a comprehensive cardiac rehabilitation programme that typically includes education on increasing physical activity and reducing sedentary behaviours with a focus on increasing patient understanding of the benefits of physical activity and empowering the patient to make behavioural adjustments during their recovery. This should be complemented by education on risk factor management, medications and coping with psychological responses to the patient’s condition.	**Physicians** **Allied health professionals (e.g. nurses, physiotherapists, exercise physiologists)** **Public health professionals** **Professional societies for all of the above** **Civil society organizations**

**Multi-component workplace programmes: Implement workplace health programmes that include educational, environmental and policy interventions** in a cohesive programme that meets the needs of workers	Implement multi-component workplace physical activity programmes, especially for sedentary occupations, with the following elements: Education regarding benefits of physical activity and access to programmes. Supportive facilities including end-of-trip facilities, safe lock-up for bicycles. Supportive environments for walking and cycling to work. Supportive office design, furniture and ergonomics to reduce prolonged sedentary time and promote incidental and light physical activity. Provision of on-site exercise facilities, showers and/or access to nearby facilities in the neighbourhood. Workplace programmes to support physical activity for all employees. Accommodate physical activity for remote-area workplaces, work-at-home, and hybrid work arrangements. Pre-tax salary sacrifice or discount schemes for purchase of bikes and scooters for commuting. Employer provision of bikes (‘bike pools’) for employee use.	**Workplace management** **Occupational health and safety professionals** **Health professionals** **Trade unions and labour organizations** **Professional societies for the above**

**Active sport and recreation settings: Promote and support participation in physical activity across the life course through organized sport and recreation groups and clubs, events and programmes**	Implement a ‘sport for all’ approach that encourages enjoyable participation in sport and active recreation across the life span (WHO 2018, ISPAH 2020). Prioritise sport and active recreation initiatives that offer social, developmental and health benefits across all age and population groups. Promote policies that ensure access to public open-spaces, playing fields and nature spaces, as well as indoor and outdoor facilities for both sport and active recreation. Provide and promote programme that support participation during key life transitions and events such as leaving secondary school, changes in employment, and changes in family structure and retirement. Ensure the availability of separate opportunities for sport and recreation by sex (e.g. ensure girls from culturally diverse backgrounds have the opportunity to use public swimming pools; provide separate classes for girls and boys where appropriate).	**Governments at national, sub-national and local levels** **Sport and recreation peak bodies** **Sport and recreation professionals** **Professional societies**

**Active programmes for older adults: Support healthy and active ageing through the provision of accessible physical activity programmes and supportive environments and settings**	Keeping people physically active as they age requires provision of appropriate environmental and social support, including in particular, walkable communities and accessible outdoor spaces that facilitate social engagement. Due to the prevalence of social isolation and loneliness in older adults, provision of programmes with an accent on social connection. This will also enhance sustained participation (Atkins 2019). Enhance training and competencies in health professionals and primary-care providers to support delivery education and referral to age-appropriate, accessible and affordable physical activity programmes. Provide supportive planning and design policies that support older adults’ physical activity and wellbeing, across scales from metropolitan planning to local design of neighbourhoods and housing options.	**Allied health professionals** **Physicians** **Aged care institutions**

**Active community-based programmes: Provide programmes and a supportive environment in neighbourhoods and settings close to home**	Ensure local funding for programmes and built environment solutions that meet community needs and meet diverse geographic and cultural requirements. Consult local communities regarding physical activity initiatives to ensure they are delivered in ways that meet the specific needs of communities. Provide increased resources to local government to better enable its critical role in the design and delivery of local programmes and environments that support physical activity. Develop capacity in local community agencies to develop and deliver physical activity programmes. Conduct local physical activity events or incorporate physical activity opportunities into existing events.	**Local governments** **Community agencies** **Health professionals** **Civil society organizations**

**Whole-of-community initiatives: Tackle physical activity at multiple levels, including media, settings-based programmes and environmental supports. This approach** acknowledges that a combination of these approaches is more effective than approaches in isolation	Community-wide initiatives that provide opportunity for whole-of-government leadership and cross-sectoral action, for example on active transport or healthy cities that include focus on physical activity. Community-wide initiatives are cited as a ‘best buy’ by the WHO as follows, “*Implement community-wide public education and awareness campaign for physical activity which includes a mass media campaign combined with other community-based education, motivational and environmental programmes aimed at supporting behavioural change of physical activity levels*.” (WHO 2017). Combine approaches including policy, environment and programmes are more effective to increase population levels of physical activity. These approaches target different settings, actors and strategies to change physical activity behaviour (Baker PRA et al., 2015; Bekemeier B et al., 2018). Combine built environment infrastructure, with media and information campaigns, has been shown to increase walking and cycling behaviour (Goodman A et al., 2014; Panter J et al., 2016).	**Government departments at national and sub-national levels** **Local governments** **Community agencies** **Health professionals** **Civil society organizations**


**Figure:**
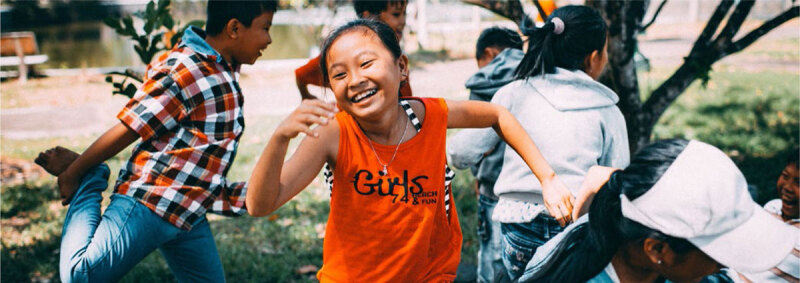

**Case study: United Kingdom**

**creating active schools**

Underpinned by behaviour change theory, the Creating Active Schools (CAS) framework identifies the ingredients required to create and promote systems change in schools by addressing the organisational structure and culture within and beyond schools. The framework seen here shows the multiple components which will facilitate whole school physical activity implementation. A video outlining the development process for the Creating Active Schools framework can be used to find out more about the rigorous process undertaken to develop the framework.

The framework alone will not be enough to influence practice. The Creating Active Schools Improvement Tool has been developed to help schools identify their own school priorities in relation to physical activity. The tool also supports schools in systematically modifying their existing structures to create positive change.

The tool starts with a “profile analysis” – requiring a school to reflect on its current provision in relation to policy, environments, stakeholders and opportunities, and identifying underserved components. This is supported by continuing professional development (CPD) e-learning. The CPD is part of the school improvement tool and is mapped to the profile, so that it directly supports the specific progress area required. While CAS was developed within a specific UK context, its flexible nature allows replication elsewhere and we would welcome others to consider how it may be used and/or adapted in different country contexts. Teachers and pupils have been instrumental in the process and have told us about the implementation of CAS and the impact it’s had on them.

**Figure:**
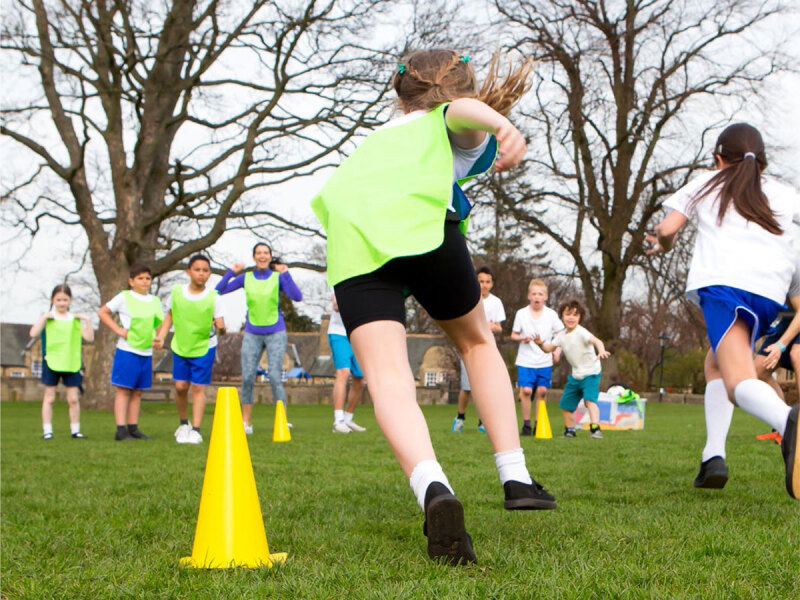

**WHO Strategic objective**

**CREATE ACTIVE SYSTEMS – Governance and policy enablers**

A systems approach ensures leadership that emphasises the integration of the multiple approaches in a physical activity action plan. It recognises that a comprehensive approach is more likely to be effective. Systems approaches can help ensure strong leadership, governance, funding streams, a capable workforce, strong institutions and multi-sector partnerships to support delivery at scale. Investment in systems approaches to noncommunicable disease prevention and control, and the promotion of physical activity, are needed to close the evidence/implementation gap and achieve UN and WHO targets (Shilton & Robertson 2018; WHO CSWG on NCDs, 2021).

**Table 10:** Recommended actions to create active systems.


RECOMMENDED ACTIONS TO CREATE ACTIVE SYSTEMS	WHAT WORKS?	KEY ACTORS

**Physical activity polices and action plans: Implement National Physical activity actions plans**, based on, and adapted from, the framework in the WHO GAPPA	A key feature of systems approaches is to embed physical activity in policy documents and plans inside and outside the health sector. National Physical Activity Plans (NPAP) provide the opportunity for countries to identify and prioritise initiatives based on local needs. The WHO GAPPA, as well as civil society resources such as this WHF policy brief, provide options for an effective community-wide response to physical inactivity. It is important that National Physical Activity Action Plans are complemented by system supports for implementation at scale. These include strengthened institutions, a skilled and strengthened workforce, sustainable financing to support implementation, mechanisms for cross-sector collaboration, meaningful engagement with civil society and communities, and investment in research and evaluation (Based on: Shilton & Robertson 2018; WHO, CCWG on NCDs, 2021).	**National and sub-national governments** **Political leaders** **Health Ministries**

**Strengthened research and evaluation: Establish support and funding for physical activity research and evaluation**	Increased investment in physical activity research and knowledge-translation to inform best investments for physical activity, cost effectiveness, and impacts of interventions on physical activity, and other health and- non-health outcomes. Strengthen research training and capacity building, especially in low- and middle-income countries and settings. Develop and sustain partnerships between government and non-government sectors as well as appropriate investment from private actors to expand physical activity research capability and impacts.	**National governments and health ministries** **Research funding agencies** **Academic institutions** **Cross-sector professionals and their professional societies**

**Strengthened data, surveillance and accountability for delivery: Strengthen and support physical activity data systems** which are vital for monitoring progress in attainment of physical activity guidelines and objectives, and for ensuring accountability for delivery	Strengthen investment in monitoring and comprehensive surveillance to ensure accountability. Develop or strengthen national data systems and measures for physical activity, and supportive policy and environment changes. Implement regular population monitoring with special focus on priority populations that are most at risk for inactivity. Ensure data collection across all ages and multiple domains. Develop objective measures and digital technologies to increase reach and accuracy of surveillance. Monitor implementation of national physical activity plans to ensure accountability for delivery.	**National governments an health ministries** **Research institutes and monitoring agencies** **Academic institutions**

**Escalate advocacy: Support and mobilize advocacy for physical activity** to influence political commitment, policy support and systems support for physical activity	Ensure research and data are used to support the urgency for change. Ensure advocacy is directed across sectors and across the life-course. Build partnerships to enhance the reach and effectiveness of advocacy. Implement a strategy mix across politics, media, professional engagement and community mobilization to build a sociocultural movement around active living. Deliver advocacy training to build capacity for physical activity advocacy across sectors.	**Civil society agencies** **Researchers and academics** **Health professionals** **Related non-health professionals (see above)** **Cross-sector professional societies**

**Strengthen financing: Ensure government and other funding for physical activity is allocated at a sufficient level to support and sustain effective delivery** of comprehensive national physical activity plans	Advocate to governments to secure allocation of sustainable financing to support delivery of national physical activity plans. Identify additional innovative sources and mechanisms to support sustainable financing, including taxes, levies, foundations, public-private partnerships and other funders.	**Health Ministries** **Finance ministries** **Foundations** **The private sector**


## Role of the world heart federation and its members

### Advocacy and leadership in global physical activity is an important strategic responsibility for the WHF

The WHF and its members across the world can make a vital contribution to re-prioritising physical activity and engaging members and stakeholders in global and local advocacy to ensure robust policies and delivery.

Leadership in physical activity also presents an opportunity for the WHF and member organisations to fill an important gap in civil society leadership in this space, in partnership with other non-government organisation, relevant professional societies, governments and other actors.

A number of Heart foundations and associations have already shown policy leadership in providing guidance on implementation of effective physical activity interventions. The National Heart Foundation of Australia *Blueprint for an Active Australia* (Third Edition) (Heart Foundation 2019) summarises, in the Australian context, compelling evidence for action to increase physical activity. The Blueprint is written for policy-makers who have an influence on national-level policies, as well as for researchers, academics and other stakeholders who seek to advocate for policy change and robust implementation. The Blueprint provides evidence and action-oriented guidance across thirteen action areas. It is a good example of a comprehensive source for physical activity planning.

The European Heart Network has developed a position paper on physical activity polices for cardiovascular health (EHN, 2019). This report presents a review of the role of physical activity in preventing and treating cardiovascular disease across Europe. It aims to provide a concise summary of recent knowledge, based on the most recent systematic reviews, meta-analyses and also scientific and policy summary statements. It is written primarily for policy-makers who have an influence on European or national-level policies influencing physical activity.

Recommendations in these and other WHF member organization positions, policies, blueprints and guidance on physical activity, as well as this WHF Physical Activity Policy Brief, need to be mobilised on a global scale. In addition, case studies of policies and programmes from around the world, in low, middle-income and high-income settings, provide a solid platform for global and local advocacy.

National Heart Foundations, Associations and Societies are well placed to maximise benefits from this WHF Physical Activity Policy Brief, responding to the unique circumstances of their country. Due consideration is needed for national adaptation of initiatives, taking into account community needs, culture, geography and the social and economic determinants of inactivity. This is relevant in all communities, but particularly in low-resource circumstances in low- and middle-income countries.

**Figure:**